# Nitric oxide as a regulator of *B. anthracis* pathogenicity

**DOI:** 10.3389/fmicb.2015.00921

**Published:** 2015-09-02

**Authors:** Taissia G. Popova, Allison Teunis, Haley Vaseghi, Weidong Zhou, Virginia Espina, Lance A. Liotta, Serguei G. Popov

**Affiliations:** ^1^National Center for Biodefense and Infectious Disease, College of Science, George Mason University, Manassas, VAUSA; ^2^Center for Applied Proteomics and Molecular Medicine, College of Science, George Mason University, Manassas, VAUSA; ^3^University of North Carolina at Chapel Hill, Chapel Hill, NCUSA

**Keywords:** anthrax, nitric oxide synthase, lymph node, proteome, nitric oxide

## Abstract

Nitric oxide (NO) is a key physiological regulator in eukaryotic and prokaryotic organisms. It can cause a variety of biological effects by reacting with its targets or/and indirectly inducing oxidative stress. NO can also be produced by bacteria including the pathogenic *Bacillus anthracis*; however, its role in the infectious process only begins to emerge. NO incapacitates macrophages by S-nitrosylating the intracellular proteins and protects *B. anthracis* from oxidative stress. It is also implicated in the formation of toxic peroxynitrite. In this study we further assessed the effects of *B. anthracis* NO produced by the NO synthase (bNOS) on bacterial metabolism and host cells in experiments with the bNOS knockout Sterne strain. The mutation abrogated accumulation of nitrite and nitrate as tracer products of NO in the culture medium and markedly attenuated growth in both aerobic and microaerobic conditions. The regulatory role of NO was also suggested by the abnormally high rate of nitrate denitrification by the mutant in the presence of oxygen. Anaerobic regulation mediated by NO was reflected in reduced fermentation of glucose by the mutant correlating with the reduced toxicity of bacteria toward host cells in culture. The toxic effect of NO required permeabilization of the target cells as well as the activity of fermentation-derived metabolite in the conditions of reduced pH. The host cells demonstrated increased phosphorylation of major survivor protein kinase AKT correlating with reduced toxicity of the mutant in comparison with Sterne. Our global proteomic analysis of lymph from the lymph nodes of infected mice harboring bacteria revealed numerous changes in the pattern and levels of proteins associated with the activity of bNOS influencing key cell physiological processes relevant to energy metabolism, growth, signal transduction, stress response, septic shock, and homeostasis. This is the first *in vivo* observation of the bacterial NO effect on the lymphatic system.

## Introduction

*Bacillus anthracis* is a soil microbe which is highly pathogenic for both humans and many animal species. It is a causative agent of anthrax which can be manifested in three major forms depending on the initiation (challenge) route: cutaneous, gastrointestinal, or inhalational. For each form, progression of the disease may result in the systemic spread of bacteria followed by septic shock and death. Inhalational anthrax has the highest mortality rate and is almost invariably lethal when left untreated (Holty, 2006). Current therapies against anthrax infection are limited to administration of antibiotics which have reduced efficacy at the late stage of disease. During the year 2000 anthrax letter attack the mortality rate was 45% even after the antibiotic therapy (Spencer, 2003). It is currently accepted that the novel approaches to treat anthrax should target not only the proliferation of bacteria but also the activity of toxic virulence factors unaffected by antibiotics (Beierlein and Anderson, 2011). However, the full spectrum of these factors acting at different stages of infection and culminating in the host death remains to be incompletely understood.

Early theories of anthrax implicated high bacterial proliferation and metabolic products as major contributors to the virulence of the microbe but were overshadowed by the discovery of two protein toxins: the Lethal Toxin (LT) and the Edema Toxin (ET) (Moayeri and Leppla, 2009). The toxins’ genes are located on the plasmid XO1. Both LT and ET contain the pore-forming component called Protective Antigen associated with the catalytic subunits, Lethal and Edema Factors, respectively. LT is a specific metalloprotease that cleaves mitogen-activated protein kinases in the host cells. ET is a calcium- and calmodulin-dependent adenylyl cyclase that converts cytosolic AMP to cyclic AMP. Both toxins were reported to be cytotoxic to certain cell types such as macrophages and lethal upon administration to experimental animals (albeit at relatively high doses; Goossens, 2009). Therefore early studies considered LT and ET as the primary causes of *B. anthracis* virulence. However, challenged animals display a wide range of sensitivity to the toxins depending on the particular strain or species (often without a direct correlation with the sensitivity to infection; Heninger et al., 2006; Lowe and Glomski, 2012). Research during the last decade shifted understanding of the toxins’ major biological function from direct killing to modulation of the host innate response helping bacteria to initiate and propagate the infectious process (Tournier et al., 2009; Guichard et al., 2012; Lowe and Glomski, 2012).

Anthrax displays remarkable histopathological features such as massive hemorrhages and tissue damage (Inglesby et al., 2002) which cannot be fully explained by the actions of LT and ET. A number of membrane-damaging and hemolytic factors, including phospholipases and the pore-forming toxin anthrolysin O, can be induced under anaerobic conditions to cause permeabilization and ultimate lysis of different cell types (Klichko et al., 2003; Popova et al., 2011). The membrane damage accompanied by the shedding of proteoglycans from the cell surface can result in the loss of tissue integrity and changes in the intercellular communication among other pathogenic consequences (Popova et al., 2006).

We recently proposed a novel mechanism of *B. anthracis* toxicity based on coordinated activity of anthrolysin O and bacterial metabolic products in conditions modeling the environment of the hypoxic host tissue. We found that the cultured epithelial cells were partially protected from the pathogenic effect of bacterial culture supernatants (sups) by chemical compounds causing decomposition of the peroxynitrite (ONOO^-^), a highly toxic substance formed in the fast reaction of superoxide radical (O_2_^-^) and nitric oxide (NO) (Popova et al., 2011; St John et al., 2013).

The appearance of peroxynitrite indicated that the mechanism of toxicity was associated with oxidative stress which can take place when actively respiring host cells or bacteria are exposed to conditions (such as reduced oxygen availability, change in pH, etc.) that abruptly affect the electron transport chain. In such conditions a premature leakage of electrons to oxygen from the hampered electron transport can result in generation of superoxide radical (Imlay, 2003; Mols et al., 2009, 2010, 2011). The latter can be quickly recombined with NO to give rise to peroxynitrite (Radi et al., 2001).

Nitric oxide can be produced by both the host cells and bacteria. In the host cells, NO is a product of reaction catalyzed by mammalian NO synthase (mNOS) which exists in three isoforms (endothelial, inducible, and neuronal). NO produced by mammalian cells is involved in several physiological processes as a signaling molecule (Foster et al., 2009). Bacteria can produce NO from a variety of pathways, many of which are not dependent on NOS. NO production by bacterial species has been traditionally seen as a product of denitrification (Sobko et al., 2005, 2006; Tiso and Schechter, 2015). In this process bacteria produce NO as an intermediate product of nitrate reduction to N_2_O and ammonia or N_2_ with the purpose of acquiring energy or balancing the redox state during anaerobic respiration (Crane et al., 2010). However, several Gram-positive bacteria, including *Bacillus*, *Staphylococcus*, and *Deinococcus* species, were shown to possess bacterial analogs of mammalian NOS (bNOS; Adak et al., 2002; Sudhamsu and Crane, 2009; Crane et al., 2010; Filippovich, 2010).

The bNOS-mediated NO was implicated in the protection of bacteria against oxidative stress within host phagocytes (Gusarov and Nudler, 2012), a variety of antibiotics (Gusarov et al., 2009) and other stress factors such UV irradiation (Patel et al., 2009). It was suggested that bacterial NO functions to decrease the number of available reduced thiols that would participate in formation of the DNA-damaging hydroxyl radical (Sudhamsu and Crane, 2009). In addition, NO activates catalase to help bacteria mitigate the oxidative stress by converting hydrogen peroxide to water and oxygen. Bacterial NO can also contribute to the direct killing of macrophages engulfing bacteria by *S*-nitrosylation of bioenergetic-related proteins within mitochondria (Chung et al., 2012). Nitroso-proteomic analysis coupled with a biotin-switch technique demonstrated that *B. anthracis* Sterne strain can produce NO during early stage of infection ultimately resulting in S-nitrosylation of proteins in *B. anthracis*-susceptible RAW264.7 macrophages. For each target respiratory chain enzyme complex tested (complex I, complex III, and complex IV) the infection by *B. anthracis* Sterne caused enzyme inhibition. Nω-nitro-L-arginine methyl ester, an NOS inhibitor, reduced *S*-nitrosylation and partially restored cell viability evaluated by the intracellular ATP levels in macrophages. Further evidence supporting protection of bacteria by bNOS was obtained in experiments with bNOS-knockout (ΔNOS) *B. anthracis* strain. When injected into mice, the bacteria demonstrate decreased virulence compared to the isogenic wild-type Sterne strain (Shatalin et al., 2008). In contrast, deletions of inducible and endothelial NOS isoforms in the host cells have no effect on susceptibility of mice to *B. anthracis* and LT (Kalns et al., 2002; Moayeri et al., 2009).

In this study we report further characterization of the *B. anthracis* bNOS role using the ΔNOS strain. We found that bacterial NO influences many facets of *B. anthracis* physiology including regulation of growth, phosphoprotein signaling and toxicity induced in the cultured host cells. In the experiments with spore-challenged mice we also analyzed the effect of NO generation by bNOS on the composition of the proteome in the lymph nodes (LNs) which are the early targets involved in the establishment and propagation of *B. anthracis* infection. This information expands our knowledge of *B. anthracis* virulence mechanisms necessary for the development of better therapies against anthrax.

## Materials and Methods

### Reagents

All reagents were from Sigma–Aldrich. Bovine serum albumin (BSA) was of >98% purity, essentially free from fatty acids and globulin. All cell culture reagents and formulated media were purchased from Mediatech, Inc., Manassas, VA, USA. Complete Serum-Free Medium (CSFM) is a proprietary serum-free and low-protein formulation based on DMEM/F12, RPMI 1640, and McCoy’s 5A. It does not contain any insulin, transferrin, cholesterol, growth, or attachment factors. The manufacturer indicates that the medium contains trace elements, high-molecular-weight carbohydrates, extra vitamins, a high-quality BSA (1 g/l). Our analysis shows that it contains *ca.* 300 μM nitrate. Griess test (Life Technologies) was used for nitrite/nitrate measurements according to manufacturer’s protocol. Antibodies against rabbit IgG (horseradish peroxidase-linked) and phosphorylated AKT (Ser473) were from Cell Signaling Technology.

### Bacterial Culture Supernatants (sups)

The Sterne strain of *B. anthracis* (34F2) is fully toxinogenic but strongly attenuated due to the lack of a polypeptide capsule. It was obtained from Colorado Serum Co. The ΔNOS strain is an isogenic derivative of 34F2 strain. It is a kind gift by Dr. E. Nudler (NY University School of Medicine, New York, NY, USA) generated by inactivation of *bnos* gene with a kanamycin (Km) cassette as described in (Shatalin et al., 2008). The primary sequence of ΔNOS DNA in the region of *bnos* determined by us for two randomly selected colonies obtained from Luria broth (LB)-agar plate containing 100 μg/ml Km is shown in Supplementary Figure. The *bnos* gene is interrupted by the insertion of a streptothricin acetyltransferase gene resulting in a frameshift. The spores were prepared as described (Popov et al., 2002). In the case of ΔNOS strain the culture medium was supplemented with 100 μg/ml Km. Bacterial culture sups were produced by inoculation of spores (to final concentration in the range from *ca.* 2 × 10^5^ to 10^6^ spores/ml) into the DMEM/F12 or DMEM supplemented with non-essential amino acids, pyruvate, glutamine, and 1 g/l of BSA unless indicated otherwise. When indicated the bacteria were grown in a preformulated serum-free medium (CSFM) from Mediatech, Manassas, VA, USA, containing among other components 1 mg/ml of BSA and about 300 μM of nitrate. The cultures were incubated at 37°C, 5% CO_2_ in 6- or 12-well tissue culture plates in static conditions or with shaking for 24 h. Bacterial growth was measured by optical density at 600 nm (OD_600_) using 96-well plates and 200 μl of suspension per well. Bacteria were removed by centrifugation and sups were supplemented with 100 μg/ml of streptomycin and 100 U/ml of penicillin to exclude growth of any contaminating bacteria. The sups were tested within a few hours after preparation.

### Cell Viability and Permeability

Primary human small-airway lung epithelial cells (HSAECs) from Cambrex, Inc., Walkersville, MD, USA were used for viability and permeability assays. The cells were seeded at density 2.5 × 10^4^/well and grown until confluent in DMEM/F12 (or DMEM) medium from Mediatech supplemented with glucose, non-essential amino acids, pyruvate, glutamate, and 10% fetal calf serum at 37°C, 5% CO_2_ using 96-well culture plates. In challenge experiments the growth medium was removed, the cells were washed three times with warm HEPES-buffered saline (HBSS) and then incubated at 37°C, 5% CO_2_ without shaking with 200 μl/well of bacterial culture sups for 2 h, unless specified otherwise. Viability of cells after challenge was routinely determined using the redox dye Alamar Blue (Resazurin) which is a water-soluble, non-toxic, fluorometric/colorimetric growth indicator. Cellular growth and metabolism reduce the dye and change its color and fluorescence. Briefly, the cells were washed with HBSS, 100 μl of Alamar Blue in CSFM were added and incubated (typically from 30 min to 1 h) with the cells until the accumulation of sufficient fluorescence from the reduced Resazurin which was measured at 530/590 nm. The viability of treated cells was calculated as the amount of fluorescence relative to the untreated controls.

Changes in the membrane integrity were measured using CellTox Green Cytotoxicity assay (Promega) according to manufacturer’s protocol. Briefly, HSAECs were exposed to the sups as described above. The cells were washed with HBSS and the appropriately diluted CellTox dye was added. Fluorescence at 485/530 nm reflecting the amount of the dye migrated into the cells was measured after 130 min of incubation at 37°C. The wells of control cells treated with the lysis buffer were used as a 100% permeability control. The permeability of treated cells was calculated as the amount of dye fluorescence relative to the lysed control.

### Effect of Sterne and ΔNOS Sups on the AKT Signaling in HSAECs

The HSAEC cells were treated with sups in 96-well plates as described above. The sups were quickly discarded and the cells were lysed with 50 μl/well of 1x SDS-PAGE loading buffer with cocktail of protease inhibitors (Pierce, IL, USA), phosphatase inhibitors (50 mM sodium fluoride, 0.2 mM sodium vanadate), 2 mM EDTA, and 6 mM DTT. Proteins were transferred to a nitrocellulose membrane using iBlot Gel Transfer Device (Invitrogen). The membranes were developed with SuperSignal West Femto Maximum Sensitivity Substrate (Pierce) and band intensities were measured with Molecular Imager ChemiDoc XRS System (Bio-Rad). The intensities of bands were calculated relative to untreated cells after densitometry using Quantity One-4.6.5 program (Bio-Rad). All measurements were done in triplicates. Each membrane was re-probed with the antibody against actin (Cell Signaling Technology) as a housekeeping protein, and the intensities of the actin bands were used to normalize the phosphorylation data.

### Animal Challenge and Extraction of Proteins from LNs

All animal procedures were approved by the George Mason University Institutional Animal Care and Use Committee. All surgeries were performed after carbon dioxide asphyxiation, and all efforts were made to minimize suffering. Female 6- to 8-week-old DBA/2J mice (Jackson Labs) received food and water *ad libitum* and were challenged with *B. anthracis* Sterne 34F2 or the bNOS knockout mutant Sterne (ΔNOS) spores (4 × 10^6^ spores in 20 μl of PBS intradermally into both hind footpads). Twenty animals were used for each strain to obtain the mortality curves. Survival of animals was monitored for 4 days. To analyze the LNs, four animals were euthanize per a time point. Thirty min before euthanasia the animals were anesthetized with isoflurane and 20 μl mixture containing 1% tracer dye Evans Blue in PBS was injected into foot pads. The popliteal LNs were surgically removed into 10% neutral buffered formalin solution for a histological evaluation and proteomic analysis as described below. Control non-infected animals received equal volume of PBS. To determine a bacterial load the spleens and LNs were homogenized on ice using frosted glass slides. The tissues were re-suspended in ice-cold PBS and plated in different dilutions onto LB agar plates. The plates were incubated at 37°C overnight. The number of colonies grown reflected the number of colony-forming units (CFUs) representing viable spores and vegetative bacterial cells.

### Mass Spectrometry (MS) Data Acquisition and Analysis

The surgically removed LNs were put on ice, trimmed of the surrounding tissue and dissected into multiple pieces with a razor blade. To extract soluble proteins the LN tissues were suspended in 100 μl of PBS containing protease inhibitors (Pierce) and finally spun at 10,000 g for 5 min to pellet tissue debris and bacteria. The supernatants containing extracted proteins from individual LNs corresponding to a particular time point were pooled and used for MS analysis. The pooled samples were dried with SpeedVac, reconstituted in 8 M urea, reduced by 10 mM DTT for 30 min, alkylated by 50 mM iodoacetamide for 30 min, and digested by trypsin at 37°C overnight. Tryptic peptides were further purified by Zip-Tip (Millipore) and analyzed by LC-MS/MS using a linear ion-trap mass spectrometer (LTQ, Orbitrap). After sample injection, the column was washed for 5 min with mobile phase A (0.4% acetic acid) and peptides eluted using a linear gradient of 0% mobile phase B (0.4% acetic acid, 80% acetonitrile) to 50% mobile phase B in 30 min at 250 nl/min, then to 100% mobile phase B for an additional 5 min. The LTQ mass spectrometer was operated in a data-dependent mode in which each full MS scan was followed by five MS/MS scans where the five most abundant molecular ions were dynamically selected for collision-induced dissociation using normalized collision energy of 35%. Tandem mass spectra were collected by Xcalibur 2.0.2 and searched against the NCBI mouse protein database using SEQUEST (Bioworks 3.3.1 software from ThermoFisher) using tryptic cleavage constraints. Mass tolerance for precursor ions was 5 ppm and mass tolerance for fragment ions was 0.25 Da. SEQUEST filter criteria were: Xcorr vs. charge 1.9, 2.2, 3.5 for 1+, 2+, 3+ ions; maximum probability of randomized identification of peptide <0.01. Protein identifications and number of identifying spectra (peptide hits) for each sample were exported using >99% confidence limit for protein identification.

The proteins identified at the particular day post challenge from LNs of naïve (day 0) and infected mice (days 1–4) were compiled in a list. For a particular protein, the number of spectral hits served as measure for comparing changes in protein abundance as described by Faca et al. (2008) for analysis of plasma proteins (Faca et al., 2008). In order to increase reliability of protein identifications, as a preliminary condition, we excluded from consideration the proteins with single spectral hits which were unique among all tested samples. The protein was considered to be up- or down-regulated by infection based on the average number of hits in infected mice at the particular time point post challenge in comparison with the number of hits corresponding to naïve mice.

For annotation analysis, GI protein accession numbers were uploaded into the DAVID (Database for Annotation, Visualization, and Integrated Discovery) informatics tool (DAVID Bioinformatics Resources 6.7; Huang et al., 2009). For GO Term (Gene Ontology) analysis we studied the Biological Process categories using the GO FAT default settings. Use of GO FAT generated more informative results than any specific GO term level. At these settings the program used a subset of GO terms depleted of the broadest terms (primarily from the top five levels of the tree) to avoid overshadowing of the more specific terms (term specificity defined on the basis of the number of child terms in the hierarchy; see DAVID website).

For functional annotation searches we set the following parameters: threshold count 3, EASE score (enrichment probability) 0.1; medium stringency for functional annotation clusters. Enrichment values (for GO terms), enrichment scores (for annotation clusters), and statistical determinants (for *p*-values and Benjamini coefficients) are those calculated by DAVID software. The Group Enrichment Score is a geometric mean (in -lg scale) of member’s Fisher exact test *p*-values in a corresponding annotation cluster, where each member’s *p*-value reflects the probability of enrichment for a particular gene in a given gene list. The Benjamini coefficients are Benjamini-Hochberg-corrected *p-*values adjusted for multiple comparisons to lower the family-wise false discovery rate and thus are more conservative than Fisher exact *p*-values.

### Statistical Analysis

Prism software (GraphPad, USA) was used for statistical analysis. Results are shown as mean ± 95% confidence intervals. Data were analyzed by two-tailed one-way ANOVA, *p* < 0.05 was considered statistically significant.

## Results

### bNOS Mutation Shows Reduced Growth and Increased Denitrification Rates in Aerated Conditions

**FIGURE 1:**
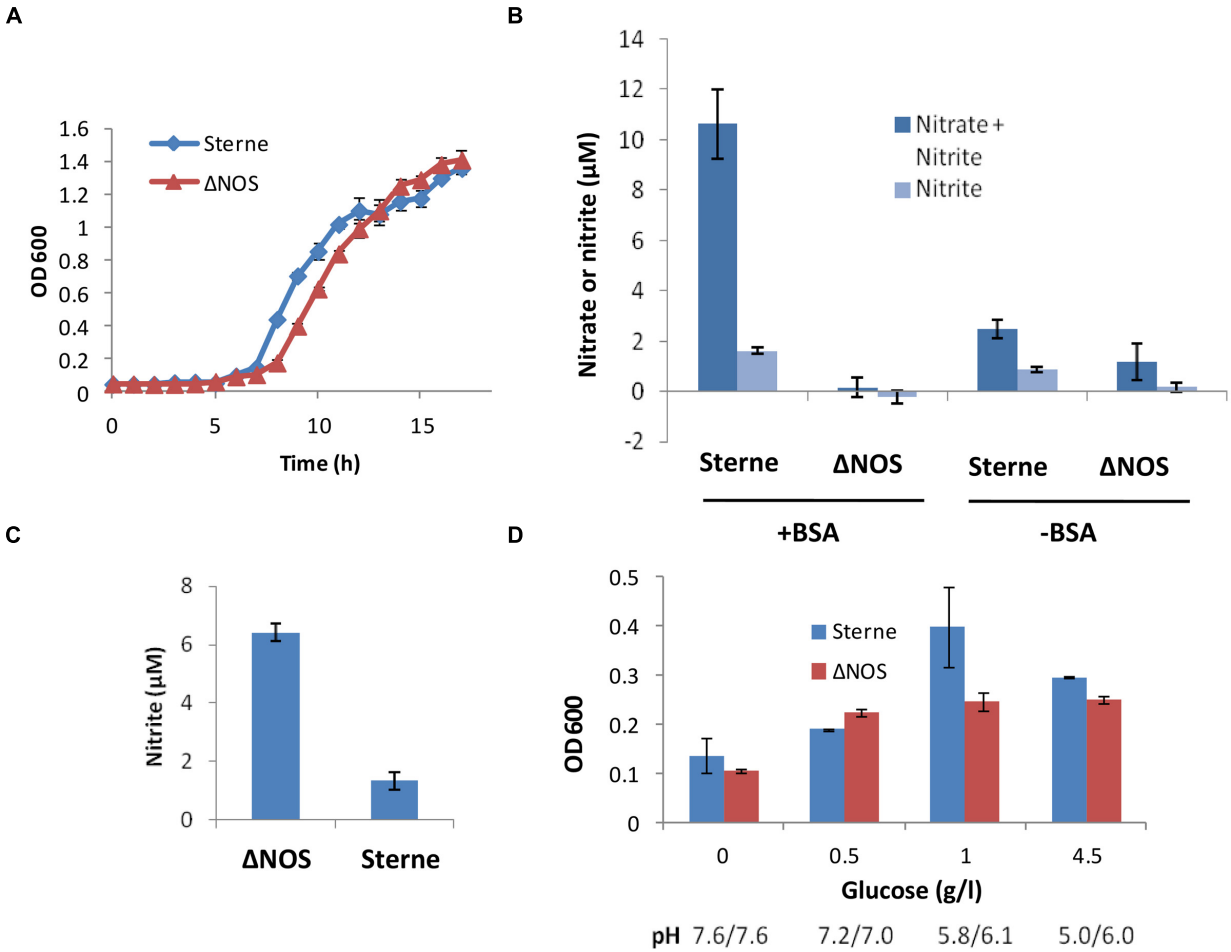
**Growth properties of Sterne and ΔNOS strains **(A,D)** and accumulation of nitrate/nitrite in cultures **(B,C)**. (A)** The growth was measures as OD_600_ at 37°C and shaking at 300 r.p.m. after inoculation of Luria broth (LB) medium with equal amounts of bacteria from overnight cultures. **(B)** Nitrate and nitrite were measured using a colorimetric Griess test (Life Technologies) in the sups of cultures grown in 12-well plates upon inoculation of 3 × 10^6^ spores per ml of DMEM/F12 medium supplemented with 1 mg/ml Bovine serum albumin (BSA), with shaking in at 5% CO_2_, 37°C for 18 h. The background values corresponding to the control wells with medium were subtracted from those with sups. The OD_600_ of the Sterne vs. ΔNOS cultures measured in 200 μl volumes using 96-well plate were 0.48 vs. 0.36 (without BSA) and 1.07 vs. 0.77 (with BSA), respectively. No substantial acidification of the medium was detected, demonstrating the absence of acidic fermentation. **(C)** The appearance of nitrite as a result respiration on nitrate in the Complete Serum-Free Medium (CSFM) culture medium containing 300 μM of nitrate was measured using Griess test. Cultures were grown at 37°C and shaking at 300 r.p.m. for 17 h upon inoculation of 3 × 10^6^ spores per ml of CSFM. **(D)** The growth of static cultures at different glucose concentrations in the DMEM medium at 5% CO_2_, 37°C for 24 h. The final pH values of the sups are shown below the corresponding bars. The error bars in all panels indicate 95% confidence intervals of triplicate measurements.

Cultivation of the ΔNOS and the isogenic Sterne strains in the standard Luria broth (LB) medium under aerobic conditions showed that the presence of bNOS was not essential for growth of the oxygen-respiring bacteria in rich medium. However, the mutation considerably delayed the growth (**Figure 1A**). To test if the bNOS represented a sole source of NO, we analyzed the amount of tracer end products, nitrate and nitrite, which form quickly upon spontaneous oxidation of NO. For this purpose we used a cell culture medium DMEM/F12 which is more relevant to the environment of the infected host than common microbiological media. It has a defined composition containing only a low concentration of nitrate (1.7 μM) which does not interfere with detection of the released NO. It is also known to support bNOS expression under aerobic and microaerobic conditions (St John et al., 2013). **Figure 1B** shows that upon aeration the Sterne strain produced detectable amounts of nitrite/nitrate, but in the case of the ΔNOS mutant strain the amount of nitrite/nitrate wasn’t statistically different from the background. The accumulation of nitrate/nitrite by Sterne strain, but not the mutant one, increased considerably when the medium was supplemented with BSA, in agreement with the previously reported NO-trapping effect of BSA (Rafikova et al., 2002; St John et al., 2013). In both cases (with and without BSA) the mutant strain showed a retarded growth compared with the Sterne strain (see **Figure 1**). These results confirmed that the bNOS deletion resulted in the loss of NO production under aerobic conditions and demonstrated that absence of NO hampered bacterial metabolism and growth.

Next we tested if the ΔNOS mutant was able to generate NO by the alternative route through reduction of nitrate in the medium containing high amount of nitrate. The first step of this process, a conversion of nitrate to nitrite by the microbial respiratory system, is known to be suppressed by oxygen and typically takes place under hypoxic or anoxic conditions (Rosenfeld et al., 2005; Bueno et al., 2012). In agreement with this, Sterne bacteria grown in aerated CSFM (serum-free medium containing ∼300 μM nitrate) did not respond to the presence of nitrate and produced no additional nitrite compared with DMEM/F12 medium (**Figure 1C**). However, the mutant strain showed increased denitrification in spite of the presence of oxygen, indicating that the absence of bNOS interfered with proper oxygen sensing by bacteria.

### ΔNOS Mutant is Deficient in Production of Acidic Fermentation Products and Cytotoxicity upon Growth in Microaerobic Conditions

In the microaerobic conditions of reduced oxygen availability which takes place during static growth the *B. anthracis* cultures demonstrate LT-independent cytotoxicity (Popova et al., 2011; St John et al., 2013) undetectable in aerated cultures. bNOS was previously implicated in this effect (St John et al., 2013) and therefore we tested the properties of the ΔNOS mutant in microaerobic conditions. Similar to the aerated conditions, the growth of the ΔNOS strain in static cultures was decreased compared to Sterne strain (**Figure 1D**). As a characteristic feature of anaerobic fermentation the growing cultures demonstrated acidification of the medium (resulting in a partial reduction of growth at high glucose). However, ΔNOS produced fewer amounts of acidic products compared to Sterne, indicating inhibition of the anaerobic glycolysis in the absence of bNOS. The acidification was directly relevant to glycolysis because increased concentrations of glucose stimulated acidification and growth while the cultures without glucose grew poorly (**Figure 1D**). No release of nitrate or nitrite was detected in either strain in the DMEM/F12 or CSFM above the sensitivity level of the Griess detection assay (>1 μM; data not shown). This result was expected because nitrate and nitrite can be respired in microaerobic conditions. Overall, cultivation experiments demonstrated that the bNOS-produced NO was required to regulate growth and response to the redox conditions in the environment.

**FIGURE 2:**
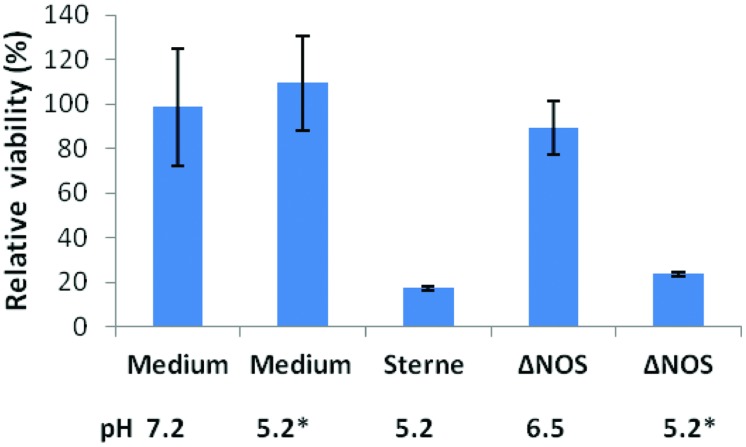
**Cytotoxic effect of Sterne and ΔNOS static culture sups.** Bacteria were grown in DMEM (10 ml, 24 h, 37°C, 5% CO_2_) in static conditions and the culture sups were prepared. The monolayers of human small-airway lung epithelial cells (HSAECs) were exposed to the bacterial sups for 2 h and the viability of the cells was determined using Resazurin dye as described in Section “Materials and Methods”. The final pH values of the sups and control medium are shown. Asterisks indicate the sups titrated to the pH 5.2 of Sterne sup after cultivation. The error bars indicate 95% confidence intervals of triplicate measurements.

**FIGURE 3:**
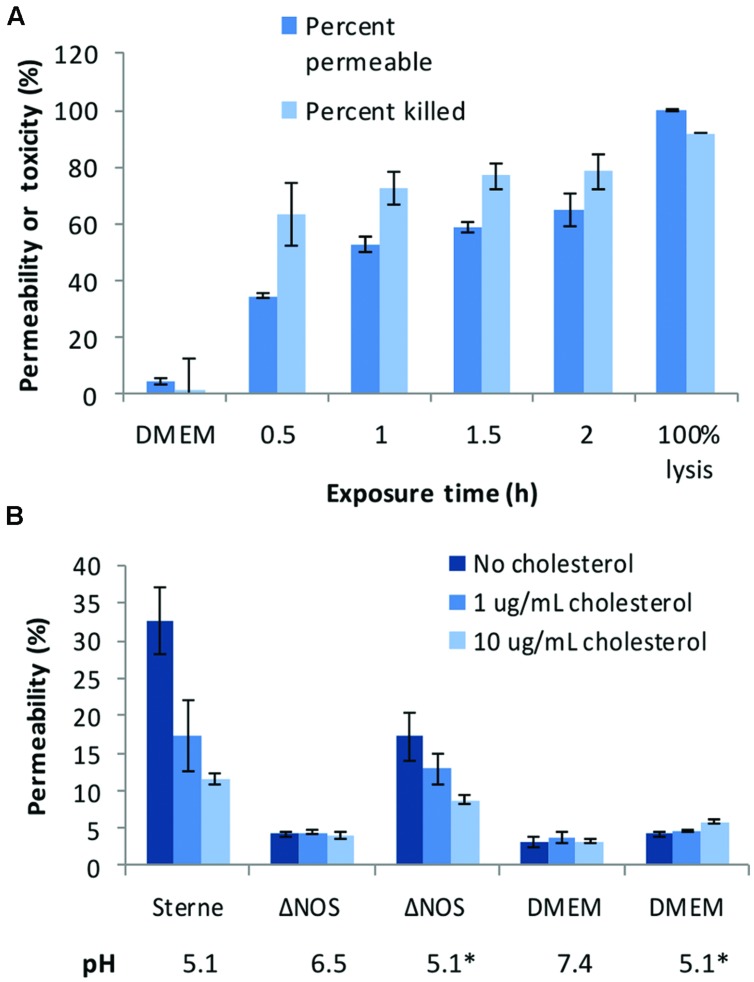
**Cell-permeabilizing effects of Sterne and ΔNOS static culture sups. (A)** Toxicity of the Sterne sup toward lung epithelial cells (HSAECs) correlates with increased permeability. HSAECs were exposed to the static culture sups for the indicated periods of time and the viability and permeability were tested as described in Section “Materials and Methods”. **(B)** Comparative effect of ΔNOS and Sterne sups after a 30-min exposure of HSAECs in conditions equal to **(A)**. Cholesterol added to the culture medium reveals presence of the pH-dependent permeabilizing factor(s). Asterisks indicate the sups and medium titrated to the pH 5.1 of Sterne sup after cultivation. Error bars show 95% confidence intervals of triplicate measurements.

Decreased acidification by the ΔNOS mutant in comparison with Sterne was associated with the reduction in the toxicity of culture supernatants (sups) toward cultured host cells. **Figure 2** shows the results of the lung epithelial cell viability assay carried out using the Alamar Blue dye reflecting the reducing capacity of viable cells. The mutant cultures were less toxic and less acidic than the Sterne ones. In order to take into account the effect of pH the ΔNOS sups were titrated using HCl to the pH values of corresponding Sterne sups. The acidified ΔNOS sups increased their toxicity, but in control incubations the acidification of the unconditioned medium did not have a toxic effect (**Figure 2**). The cells were also tested for the extent of membrane permeability using the CellTox assay (Promega) after exposure to sups (**Figure 3A**). The amount of green fluorescence due to the interaction of the cyanine dye with DNA of dead or permeabilized cells was measured. In correlation with the viability assay, the ΔNOS sups were found to be substantially less active than the Sterne ones but showed increased permeabilization after acidification to the pH of Sterne sups. Cholesterol was able to partially reduce the permeabilization (**Figure 3B**) in accordance with the presence of the pore-forming cholesterol-sensitive cytolysin ALO in the sups (Popova et al., 2011). These results demonstrated that the membrane-damaging bacterial factor(s) was (were) present in the sups of both ΔNOS and Sterne cultures; however, its full activity required a reduced pH.

### Deletion of bNOS Abrogates Pathogenic Effect of *B. anthracis* on AKT Signaling

**FIGURE 4:**
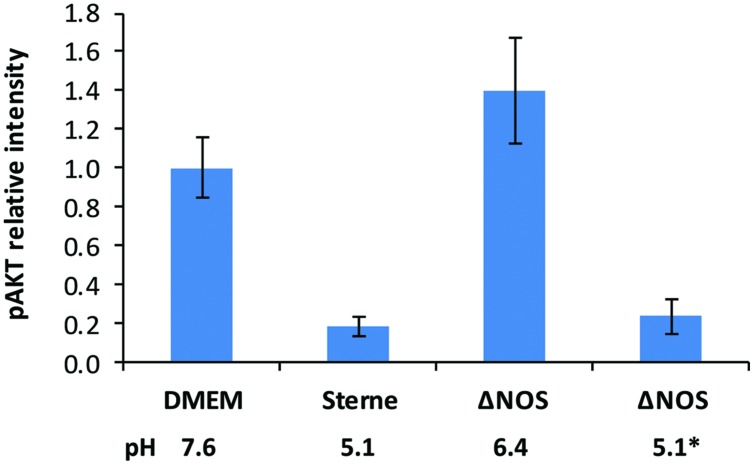
**Effect of Sterne and ΔNOS sups grown in microaerobic conditions on phosphorylation of AKT (S473) in HSAECs.** The cells were exposed for 2 h to the sups of bacterial cultures grown for 24 h in static conditions. Corresponding pH values of the sups are shown. The lysates of the cells were used for western blots with a primary rabbit antibody against phosphorylated form of AKT (S473) and a secondary anti-rabit antibody conjugated with horseradish peroxidase. The activity of peroxidase in the bands was detected using a SuperSignal West Femto chemiluminescent substrate (Pierce) and quantitated with a digital camera. To take into account the effect of pH the ΔNOS sup after cultivation was titrated from pH 6.4 to pH 5.1 indicated by an asterik. Error bars show 95% confidence intervals obtained from three independent western blots. The preparation of the lysates was repeated twice with similar results.

The reduced toxic effect of the ΔNOS mutant compared with Sterne correlated with higher phosphorylation levels of the major cell survival signaling kinase AKT (**Figure 4**). The cells were exposed to the sups of Sterne and ΔNOS strains grown in microaerobic conditions and western blot was used to detect the amount of phosphorylated AKT (S473). Relative to the control medium the Sterne sup-treated cells showed a strong inhibition of AKT phosphorylation while the ΔNOS sup stimulated AKT. However, the cells treated with the ΔNOS sup at pH reduced to 5.1 (corresponding to the Sterne sup) demonstrated a strong loss of phosphate reflecting a reduced survival signaling.

### Virulence of ΔNOS Mutant in *B. anthracis*-Susceptible Mice

The effect of bNOS on virulence was tested in susceptible DBA/2J mice which were challenged with spores into hind footpads. This inoculation route provides experimental means to direct a spore delivery to the draining popliteal, inguinal, and sub-iliac LNs with the lymph flow within minutes post challenge in order to study interaction of the lymphatic system with the pathogen during infection (Popova et al., 2014). LNs are known early targets of anthrax allowing the microbe to establish infection and disseminate to distant locations (Weiner and Glomski, 2012). One of the important physiological properties of LNs is a low oxygen pressure (Caldwell et al., 2001) which affects the bacterial metabolism and host response, and may result in the appearance of toxic products such as peroxynitrite (Radi et al., 2001).

**FIGURE 5:**
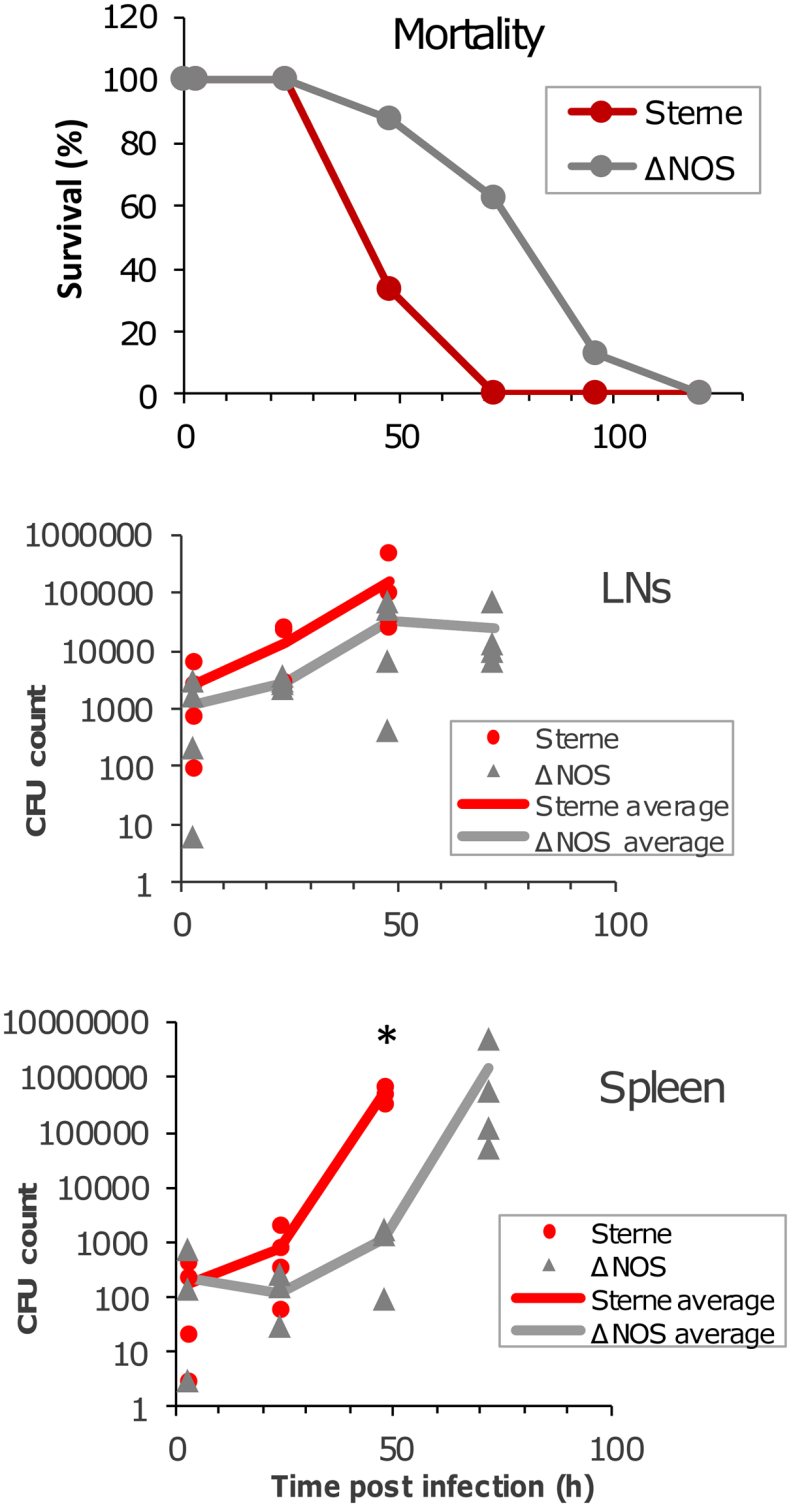
**Mortality curves and bacterial load in LNs and the spleen of spore-challenged DBA/2J mice.** Animals were challenged with the toxinogenic, non-encapsulated *Bacillus anthracis* Sterne 34F2 or ΔNOS knockout Sterne spores (4 × 10^6^ spores in 20 μl of PBS), intradermally into both hind footpads. Twenty animals were used for each strain to obtain the mortality curves. Four animals were euthanized at each time point to determine a bacterial load in the LNs and spleen. The organs were surgically removed and homogenized. The homogenates were seeded onto agar plates to determine the number of colony-forming units (CFUs). The CFU values are shown for each animal, and the lines in the panels are drawn through the average CFU values for each time point. A statistically reliable difference (*t*-test, *p* < 0.001) between Sterne and ΔNOS in the spleen at 48 h post challenge is indicated by an asterisk.

The mortality curves (**Figure 5**) show that it took the ΔNOS strain approximately 40 h longer than Sterne to reach 50% mortality. However, as in the case of Sterne, all mice ultimately died demonstrating that deletion of bNOS did not abrogate the toxicity but rather delayed it. The time course of dissemination of infectious material to draining popliteal LN and spleen was tested by seeding the organ homogenates onto the LB agar plates. At 3 h post challenge a large number of CFUs representing viable spores and vegetative cells were detected in the LNs and spleens of mice challenged with Sterne and ΔNOS strains. The amount of infectious material continued to increase steadily during infection with Sterne spores until all mice succumbed to the disease at 72 h (at this time point no survived animals could be tested). In comparison with Sterne the ΔNOS strain showed a reduced virulence correlating with a slower propagation of bacteria in the LNs and a reduced dissemination to the spleen. Although the observed difference between strains in the LNs did not reach statistical reliability, the spleen counts were reliably different at 48 h post challenge (*p* < 0.001).

### Global Proteomic Analysis of LNs in Infected Mice

**FIGURE 6:**
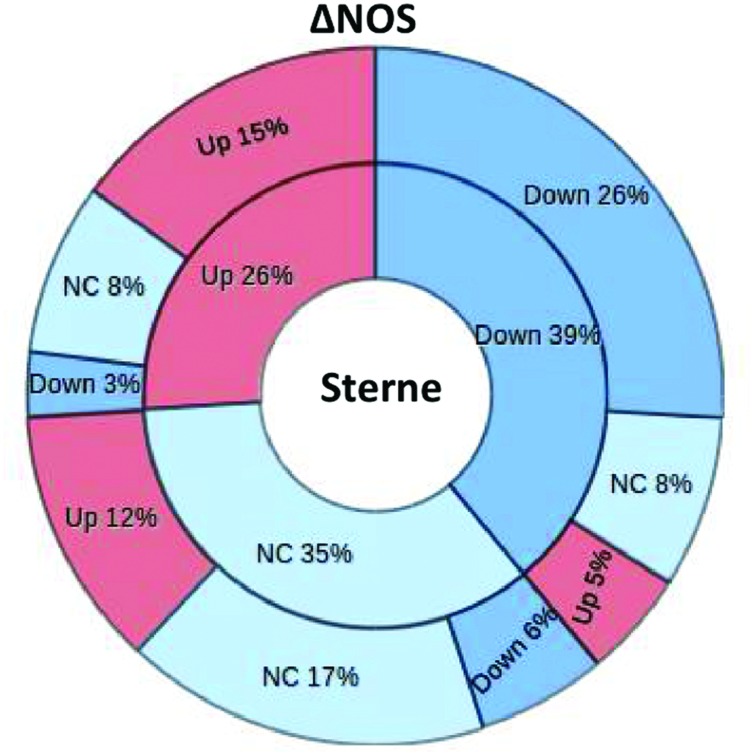
**The ΔNOS infection leads to global changes in the lymph proteome relative to Sterme one.** The segments of pie chart show the number of proteins from the total list of 581 proteins identified by MS as upregulated, downregulated, or unchanged during infection relative to uninfected mice. The inner circle corresponds to the Sterne-infected mice. The outer circle demonstrates changes of this pattern observed in ΔNOS-infected mice.

In order to assess the impact of bNOS on the infectious process we carried out a global proteomic analysis of the host response in the LNs of spore-challenged mice. The LNs were previously shown to behave as sensitive sensors responding to the presence of *B. anthracis* pathogenic factors with numerous changes in the content and levels of lymph proteins (Popova et al., 2014). After an intradermal challenge of mice with spores into hind footpads the sentinel popliteal LNs were surgically removed and soluble proteins extracted with PBS in the conditions avoiding cell lysis. The protein composition of the samples after digestion with trypsin was determined by tandem MS. The amount of spectral hits reflecting the number of matches corresponding to a particular protein was considered as a semi-quantitative measure of protein abundance (Faca et al., 2008). The lists of identified proteins from the Sterne- and ΔNOS-infected mice, as well as the control uninfected mice, were compiled and compared to each other. In the case of both strains, the animals were tested at day 2 post challenge corresponding to the onset of pre-mortal condition in Sterne-infected mice. Additionally, the ΔNOS-infected mice were tested at the pre-mortal condition at day 4 post challenge. A total of 581 proteins were identified in both experiments. Among these proteins, 39% were downregulated, 26% were upregulated, and 35% did not change their levels in Sterne infection in comparison with the control uninfected mice (**Figure 6**). The ΔNOS infection led to global changes in the lymph proteome relative to Sterne. From the 39% of Sterne-downregulated proteins, 26% remained downregulated during ΔNOS infection, while 13% showed the opposite trend (became upregulated or equal to control). Further, among the 26% of Sterne-upregulated proteins, 15% remained upregulated while 11% showed the opposite trend. Finally, among the 35% with the levels equal to controls, the ΔNOS changed expression of 18% (upregulated 12% and downregulated 6%). Overall, 42% of proteins changed their mode of expression as a result of bNOS mutation demonstrating that it had a strong impact on the interaction of *B. anthracis* with the host.

### DAVID-Based Analysis of the Host Processes Associated with bNOS Mutation

The Database for Annotation, Visualization, and Integrated Discovery (DAVID) Functional Classification tool generates a gene-to-gene similarity matrix-based shared functional annotation using over 75,000 terms reflecting biological processes from 14 functional annotation sources (Dennis et al., 2003; Huang et al., 2009). The software algorithm measures relationships among the annotation terms based on the degree of commonality in gene or protein content between two annotations to classify the groups of similar annotation. The more common proteins the annotations share, the higher chance they will be grouped together. Each annotation group (cluster) is assigned a Group Enrichment Score as the geometric mean (in -log scale) of members’ *p*-values which is used to rank their biological significance. Grouping proteins based on functional similarity can systematically enhance biological interpretation of large lists and reveal proteins involved in underlying processes. We used this tool to analyze the lists of host proteins up- and down-regulated by bNOS-expressing Sterne strain relative to the bNOS knockout ΔNOS strain. The lists were generated by subtracting the numbers of spectral hits for proteins in ΔNOS-infected mice from the corresponding numbers found in pre-mortal Sterne-infected mice at day 2 post challenge.

From the list of 157 proteins up-regulated by the presence of bNOS in Sterne relative to ΔNOS mutant, the software identified 127 proteins matching its database and categorized 104 of them into enriched processes reflecting relative contextual abundance of the proteins in the analyzed list relative to the genome-wide gene list used by DAVID as a background. In total, 124 biological processes from GO FAT database were identified. These processes were further categorized into functionally related groups using clustering algorithm. The software generated 19 clusters. The selected ones after removal of redundant GO terms are shown in Supplementary Table S1. The clusters with the highest enrichment scores were dominated by processes with GO terms reflecting chromatin assembly and disassembly, host response to wounding, blood coagulation, regulation of cell metabolism and death. The spectrum of these processes was similar to what was previously found in the case of Sterne infection (Popova et al., 2014) and likely reflects both the protective and pathogenic responses which are mediated by bNOS directly (though the effect of released NO on the host) and/or indirectly (though the effect of NO on the bacterial factors). In the case of host responses downregulated by bNOS (higher in ΔNOS-challenged mice compared to Sterne), DAVID software recognized 173 from 209 dowregulated proteins and assigned 151 of them into 280 GO processes grouped into 40 clusters. Representative examples are shown in Supplementary Table S2. The most enriched clusters included metabolic processes of carbohydrates, alcohols, lipids, glycolysis, energy derivation by oxidation of organic compounds and aerobic respiration, catabolism of tricarboxylic and other organic acids. The notable metabolic changes also include rearrangements of actin cytoskeleton, reduced responses to oxidative stress as a part of suppressed innate immunity and homeostatic imbalance associated with changes in blood coagulation, apoptotic, and protein kinase cascades. Overall, the processes in LNs indicated a broad shutdown of main cellular functions.

**FIGURE 7:**
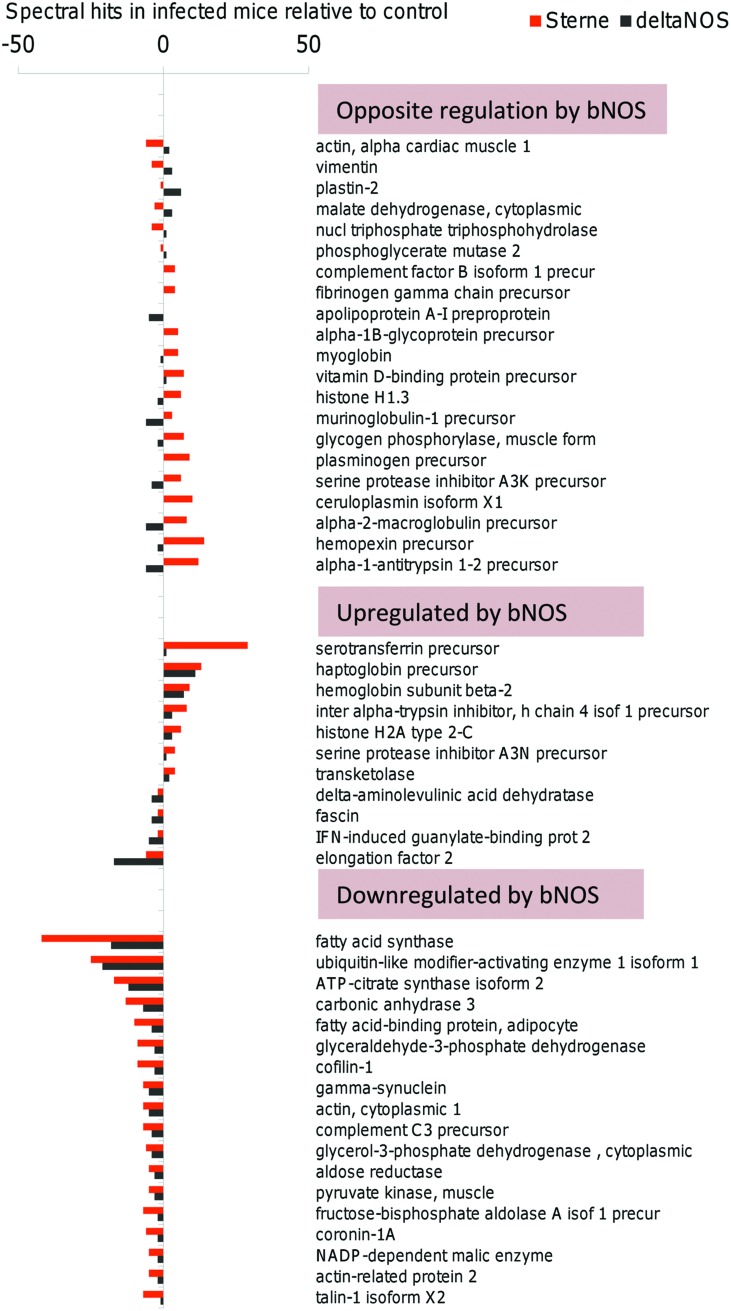
**Sterne and ΔNOS infections lead to the homeostatic imbalance of lymph proteins.** The responses which differed by three or more hits relative to uninfected control were grouped into three clusters representing the responses (i) opposite to each other between Sterne and ΔNOS strains, (ii) enhanced by bNOS-expressing Sterne, and (iii) downregulated by bNOS-expressing Sterne relative to ΔNOS.

The above analysis demonstrated high impact of bNOS-mediated bacterial factors on the major host responses associated with death of Sterne-infected mice at day 2 post challenge; however, it was not clear how these host responses related to the death of ΔNOS-infected mice which developed a pre-mortal condition at day 4 post challenge. To address this question, the lymph of pre-mortal ΔNOS-infected mice at day 4 post challenge was analyzed in comparison with the lymph from Sterne-infected mice at day 2 post challenge. **Figure 7** shows that both Sterne and ΔNOS infections lead to the homeostatic imbalance of lymph proteins (considered as a degree of deviation from the normal lymph content). To generate the figure, the full lists of proteins detected by MS were shortened to include the reliably detected responses which differed by three or more hits relative to uninfected control for a particular protein at least in one strain. The pattern of the infection-induced imbalance for the 61 selected proteins was found to be strongly influenced by the bNOS. The proteins were grouped into three clusters. In the first one the responses to Sterne and ΔNOS strains are opposite to each other and likely lead to opposite biological effects. In the second one, the presence of bNOS in the Sterne strain enhances the deviations from homeostasis through upregulation of protein levels relative to ΔNOS. Finally, the third cluster contains proteins downregulated by Sterne for which the effect of bNOS enhances downregulation relative to ΔNOS. Among the spectrum of proteins demonstrating different degrees of homeostatic imbalance in **Figure 7**, several ones induced by both Sterne and bNOS are known to be strongly associated with sepsis, tissue damage, or protective responses and therefore may be involved in the positive or negative control of lethal outcome. These are the protease inhibitors (serine protease inhibitor A3K, alpha-2-macroglobulin, alpha-1-antitrypsin-1, 2), genes involved in the iron metabolism (ceruloplasmin, hemopexin, serotransferrin, haptoglobin, hemoglobin, myoglobin), fatty acid metabolism as well as cell cytoskeleton (actin, cofilin-1, plastin-2).

## Discussion

There are only a limited number of reports on the role of bacterial NO production in the infectious process (Shatalin et al., 2008; Gusarov et al., 2009; Zeng et al., 2011; van Sorge et al., 2013). NO is a membrane-permeable gas with a short life time which can form an array of multiple interrelated redox forms (Hakim et al., 1996; Liu et al., 1998). Depending on the concentration, NO can cause two types of chemical reactions with distinct biological effects. At the micromolar level the extra-bacterial NO could behave as a potent chemical reagent causing so-called direct effects by interacting with the biological targets such as lipids, DNA, enzyme cofactors, side groups of proteins (*S*-nitrosylation and tyrosine nitration), etc., (Thomas et al., 2008). In addition, the indirect effects of NO at <200 nM can cause nitrosative and oxidative stress through formation of intermediate reactive species. The intracellular NO produced by activated macrophages can reach rather high concentrations (>1 μM) which are bactericidal.

The bNOS was previously shown to be important for *B. anthracis’*s pathogenicity during interaction with macrophages by protecting bacteria from oxidative stress and *S*-nitrosylating the macrophage intracellular proteins (Shatalin et al., 2008; Chung et al., 2012). Additional evidence of the pathogenic role of NO as a product of bNOS was obtained in the cytotoxicity studies *in vitro* when lung epithelial cells were exposed to the secreted virulence factors produced by *B. anthracis* in microaerobic conditions (Popova et al., 2011). The cells were effectively protected using specific catalysts accelerating decomposition of peroxynitrite which can be formed in the reaction of NO with superoxide radical in the conditions of oxidative stress. The peroxynitrite is extremely toxic through its radical products capable of tyrosine nitration, which is often considered as indicator of peroxynitrite formation (Pacher et al., 2007). Stearns-Kurosawa et al. (2006) reported intense tyrosine nitration in tissues of Sterne-challenged primates, but no other studies addressed the topic of NO-induced protein modifications in anthrax. *B. cereus*, a species closely related to *B. anthracis*, was also found to generate peroxynitrite under mild stress induced by reduced pH (Mols et al., 2009, 2010).

To more fully assess the role of bNOS as one of the NO sources in anthrax we decided to continue characterization of the isogenic bNOS knockout mutant of the Sterne strain. In comparative experiments with the parent Sterne strain we expected to reveal the effects of bNOS on bacterial metabolism as well as the host cells assuming that a sufficient amount of NO can diffuse from bacteria before becoming converted into stable products, nitrite, and nitrate. Current literature provides no information on the levels of NO generation by *B. anthracis* bNOS. We tested the amount of nitrite/nitrate during bacterial growth in the DMEM/F12 culture medium supplemented with BSA to retain NO in solution (Rafikova et al., 2002). Under aerated conditions the Sterne strain resulted in accumulation of more than 10 μM of nitrite/nitrate, the concentration sufficient for the direct and indirect effects of bacterial NO. This conclusion is in agreement with our previous finding of *S*-nitrosylated proteins after engulfment of *B. anthracis* spores by macrophages (Chung et al., 2012). As expected, no production of nitrite/nitrate was detected in the case of ΔNOS.

We found that the growth of the ΔNOS strain was markedly attenuated in both aerobic and microaerobic conditions of reduced oxygen indicating that NO plays an important role in the propagation of *B. anthracis*. The production of potentially dangerous NO seems to be of advantage to the microbe in spite of the fact that NO detoxification represents a metabolic burden. NO was previously shown to be involved in the regulation of switch from aerobic to anaerobic metabolism in other *Bacillus* species (Härtig and Jahn, 2012). In our experiments the regulatory role of NO was suggested by the abnormal denitrification of nitrate by ΔNOS in the presence of oxygen, indicating a deregulated oxygen-sensing mechanism which no longer shuts down aerobic respiration. Further evidence of anaerobic regulation mediated by NO came from the reduced fermentation of glucose by ΔNOS closely associated with the reduced toxicity of bacterial sups toward host cells in culture. In the presence of bNOS the bacterial sups accumulated an acidic fermentation-derived metabolite suggested in our previous study to be a succinic acid (Popova et al., 2011). The decreased pH was required for the intoxication of the target HSAECs permeabilized by the sups in the viability test. Acidification of the cell cytoplasm is known to inhibit the respiratory chain and generate reactive oxygen species killing the cell (Lambert and Brand, 2004). We suggest that in the presence of NO this process may result in the production of peroxynitrite as a secondary toxic species.

The intoxicated host cells demonstrated the increased phosphorylation of major survivor protein kinase AKT correlating with the reduced toxicity of ΔNOS sups in comparison with Sterne sups. It is plausible that the effect on host signaling may be caused by direct nitrosylation of AKT by NO blocking the phosphorylation of Ser473 residue in AKT (Slomiany and Slomiany, 2011; Asanuma et al., 2015). Further studies are required to elucidate details of this toxic mechanism, including the role of peroxynitrite.

During infection bacterial pathogens encounter host anatomical sites with low oxygen levels, a condition often exacerbated by pathological events such as ischaemia, oedema, and inflammation, triggered by the infection process. The fact that *B. anthracis* has anaerobic pathways capable of providing energy and eventually using nitrate as final electron acceptors strongly suggests that anaerobic metabolism plays an important role in anthrax. The interplay between oxygen availability and expression of virulence factors has been well documented in *B. anthracis* and other pathogenic species (Green et al., 2014). *B. anthracis* seems to be well-adapted to the hypoxic environment of LNs. Within hours after inoculation the *B. anthracis* the spores germinate in the lymphatics and give rise to large number of vegetative bacteria (Popova et al., 2014). Our results indicated that genes involved in anaerobic metabolism under the control of NO may be crucial for bacterial infection. Based on the literature data we speculate that Fnr (Fumarate and Nitrate reductase Regulator) protein (Green et al., 2014) in *B. anthracis* is the main player in the metabolic switch from aerobic to anaerobic growth similar to *B. cereus* where the anaerobiosis and the expression of enterotoxins are controlled by FNR and the two-component regulators ResDE (Zigha et al., 2007). The unphosphorylated response regulator ResD acts as an inactivator of Fnr, while ResD phosphorylated by sensor kinase ResE acts as a coactivator of Fnr (Esbelin et al., 2009). The activity of ResE is higher under anaerobic than under aerobic conditions. In *B. subtilis*, the upregulation of ResDE-dependent genes requires a sensor protein NsrR in the presence of NO. NsrR was shown to bind to the promoters of these genes and inhibit their transcription *in vitro*. NO relieves this inhibition by reacting with a (4Fe-4S)^2+^ cluster of NsrR to form dinitrosyl iron complexes (Yukl et al., 2008). This mechanism explains our observations and will serve as basis for our future exploration of the NO regulatory role in anthrax pathology.

The proteomic content of intra-nodal lymph is highly sensitive to anthrax infection and reflects a multitude of physiological changes accompanying the activity of bacterial pathogenic factors (Popova et al., 2014). Our MS-based proteomic analysis of lymph present in the infected LNs revealed numerous changes in the protein abundance associated with the activity of bNOS in Sterne relative to ΔNOS. This is the first *in vivo* observation of the bacterial NO effect on the lymphatic system. We conclude that the bNOS-generated NO has a global impact on the host cell responses with regard to the expression pattern and the levels of lymph proteins. Our bioinformatics analysis shows that bacterial NO influences key cell physiological processes relevant to energy metabolism, growth, signal transduction, stress response, septic shock, and homeostasis. The examples from the list shown in **Figure 7** include alpha-1-antitrypsin which can inhibit several serine proteases that confer inflammatory processes (Ganter et al., 1987). Some studies showed that the diagnostic value of alpha-1-antitrypsin levels can be used to identify sepsis, to differentiate bacteremia, and to assess prognosis (Buttenschoen et al., 2001). High concentrations of this protein in plasma may also be associated with the development of multiple organ failure during sepsis (Tanaka et al., 1991). Haptoglobin levels increase in patients with inflammation and infection, and this protein acts as a potent anti-inflammatory agent. It is present in the blood proteome of patients with sepsis (Kalenka et al., 2006) and may play a protective role in sepsis patients who have elevated levels of circulating cell-free hemoglobin beyond its previous description as an acute phase reactant (Janz et al., 2013). In critically ill patients with sepsis, elevated concentrations of circulating cell-free hemoglobin are independently associated with an increased risk of death. Hemopexin binds the free heme released by the turnover of heme proteins such as hemoglobin and thus protects the body from the oxidative damage that free heme can cause (Janz et al., 2013; Lin et al., 2015). Ceruloplasmin participates in antioxidant and anti-inflammatory defense (Bielli and Calabrese, 2002). Clinical trials confirmed that the serum ceruloplasmin levels may help in the diagnosis of sepsis (Chiarla et al., 2008). Fatty acid synthase upregulation by bNOS can cause activation of NLRP3 inflammasome during sepsis through the mitochondrial uncoupling protein-2 which is considered as a potential therapeutic target for inflammatory diseases (Moon et al., 2015). The pathogenic consequences of the host response to the bNOS-produced NO discussed above require further focused studies. It also seems important to determine to what extent the observed effects of NO on the host cells can be attributed to its direct or indirect chemical modifications of host targets as opposed to the regulation of bacterial metabolism in LNs and other anatomical locations harboring *B.anthracis*.

Finally, the presented data help understand pathophysiological mechanisms of NO as newly emerged virulence factor and its role in the host response to anthrax for development of effective prophylactic and treatment strategies.

## Author Contributions

Contributed to conception and design: VE, LL, TP, SP. Contributed to acquisition, analysis and interpretation of data: TP, HV, AT, WZ, SP, VE, LL. Drafted and/or revised the article: SP, TP, AT.

## Conflict of Interest Statement

The authors declare that the research was conducted in the absence of any commercial or financial relationships that could be construed as a potential conflict of interest.
