# Supplementary material for: Nitric oxide as a regulator of B. anthracis pathogenicity
Source: Front Microbiol. 2015 Sep 2;6:921. doi: 10.3389/fmicb.2015.00921 (PMC4557104; doi:10.3389/fmicb.2015.00921)
Supplement: Supplementary file 1 [file Data_Sheet_1.DOCX]

**Nitric Oxide as a Regulator of *B. anthracis* Pathogenicity**

**Taissia G. Popova ^1^, Allison Teunis ^2^, Haley Vaseghi ^3^, Weidong Zhou^2^, Virginia Espina^2^, Lance A. Liotta^2^, Serguei G. Popov^1^***

^1^National Center for Biodefense, College of Science, George Mason University, Manassas, VA, USA

^2^Center for Applied Proteomics and Molecular Medicine, College of Science, George Mason University, Manassas, VA, USA

^3^University of North Carolina at Chapel Hill

*** Correspondence:** Corresponding Author, National Center for Biodefense, College of Science, George Mason University, 10900 University Blvd, Manassas, VA, USA 20110

[spopov@gmu.edu](mailto:spopov@gmu.edu)

**1. Supplementary Figures**

1 CATTCCCTCC TTTCACCCAA AAAGTTGGTC TAGGTAAAAA TAATTTACCT

51 GAGTCAAAAA TACACCTATT TTTTATAGAA GTCAATAGTA ATCTCTTTAA

101 TTTTTTGAAA AATATTACAA TAAAAGTAAC ACTGCGACTT TCATTTGTGA

151 CTCTCGTTCT TTTTGCTTAT ACTGTAAAGT AGTAATGTTT TGTTGAAATA

201 GAAGGAGATC GTTT

[bnos] **ATG**AGT AAAACGAAAC AATTAATAGA GGAAGCGAGT

251 CATTTTATTA CGATTTGCTA TAAAGAGCTT AGTAAAGAAC ATTTCATAGA

301 AGAACGCATG AAAGAAATTC AAGCTGAGAT AGAGAAGACA GGGACATATG

351 AGCATACATT TGAAGAACTT GTTCATGGAT CGCGAATGGC ATGGCGCAAT

401 AGTAATCGAT GTATCGGAAG ACTATTTTGG AGTAAGATGC ACATATTAGA

451 TGCACGTGAA GTAAACGATG AGGAAGGTGT ATATCATGCA TTAATTCATC

501 ATATTAAATA TGCAACGAAC GACGGAAAAG TGAAACCGAC AATTACAATT

551 TTTAAGCAAT ATCAAGGTGA AGAAAATAAT ATACGAATTT ATAATCATCA

601 ATTAATTCGA TATGCAGGAT ATAAAACAGA AATGGGAGTG ACTGGTGACT

651 CTCATTCCAC TGCATTTACA GATTTTTGTC AGGAACTTGG CTGGCAAGGA

701 GAA GGC ACG [end of bnos seq]

[unknown insert] CTG’CAG’G

[streptothricin acetyltransferase, partial]

TC’GAT’AAA’ CCC’AGC’GAA’CCA’TTT’GAG’GTG’ATA’GG

751 T’AAG’ATT’ATA’CCG’AGG’TAT’GAA’AAC’GAG’AAT’TGG’ACC’TTT’ACA’GAA’TTA’C

801 TC’TAT’GAA’GCG’CCA’TAT’TTA’AAA’AGC’TAC’CAA’GAC’GAA’GAG’GAT’GAA’GAG’

851 GAT’GAG’GAG’GCA’GAT’TGC’CTT’GAA’TAT’ATT’GAC’AAT’ACT’GAT’AAG’ATA’AT

901 A’TAT’CTT’T

[streptothricin acetyltransferase deletion]

(actaccaagacgataaatgc gtcggaaaa gttaaactgcgaaaaaattggaaccggtacgct)

[streptothricin acetyltransferase, continues]

TA’TAT’AGA’AGA’TAT’CGC’CGT’ATG’**TAA**’GGATTTCAGGGGGCAA

951 GGCATAGGCA GCGCGCTTAT CAATATATCT ATAGAATGGG CAAAGCATAA

1001 AAACTTGCAT GGACTAATGC

**Supplementary Figure. DNA sequence of ΔNOS mutant in the region of *bnos* gene.** Two colonies picked up from agar plates containing 100 µg/ml Km were used for sequencing and gave identical results. The *bnos* gene sequence (capital letters) is shaded gray. The initiation and termination codons are shown in a bold font. The fragment of streptothricin acetyltransferase (capital letters, no shade) interrupts the *bnos* and results in the frame shift. Deletion in the intervening sequence is shown in low-case letters.

**Supplementary Table 1. Clusters of processes from GO FAT database associated with up-regulation by bNOS of protein levels in lymph of challenged mice**

| **ES*** | **Term** | **Count** | **FE**** | **P value** | **Benjamini** | **Genes** |
| --- | --- | --- | --- | --- | --- | --- |
| 4.8 | GO:0034622~cellular macromolecular complex assembly | 15 | 9.0 | 9.0E-10 | 7.80E-07 | 6678465, 68226431, 226443026, 119433657, 19527078, 30089710, 6678467, 34740335, 13591862, 7106439, 33859488, 12963615, 68226433, 29244126, 254588110 |
|  | GO:0031497~chromatin assembly | 8 | 13.9 | 1.5E-06 | 1.87E-4 | 68226431, 226443026, 119433657, 30089710, 13591862, 68226433, 29244126, 254588110 |
|  | GO:0034728~nucleosome organization | 8 | 13.8 | 1.6E-06 | 1.8E-4 | 68226431, 226443026, 119433657, 30089710, 13591862, 68226433, 29244126, 254588110 |
|  | GO:0065004~protein-DNA complex assembly | 8 | 13.8 | 1.6E-06 | 1.8E-4 | 68226431, 226443026, 119433657, 30089710, 13591862, 68226433, 29244126, 254588110 |
|  | GO:0006333~chromatin assembly or disassembly | 8 | 9.6 | 1.8E-05 | 0.002 | 68226431, 226443026, 119433657, 30089710, 13591862, 68226433, 29244126, 254588110 |
|  | GO:0043623~cellular protein complex assembly | 7 | 8.5 | 1.6E-04 | 0.010 | 6678465, 19527078, 6678467, 34740335, 7106439, 33859488, 12963615 |
|  | GO:0007018~microtubule-based movement | 6 | 7.8 | 1.0E-03 | 0.050 | 6678465, 6678467, 34740335, 7106439, 33859488, 12963615 |
| 1.9 | GO:0009611~response to wounding | 11 | 4.1 | 2.9E-04 | 0.017 | 160358825, 19527078, 20330802, 130503301, 218156289, 76881807, 110347406, 126518317, 257471003, 6679182, 18252782 |
|  | GO:0050817~coagulation | 4 | 7.5 | 1.6E-02 | 0.324 | 160358825, 19527078, 257471003, 18252782 |
|  | GO:0007599~hemostasis | 4 | 7.4 | 1.7E-02 | 0.327 | 160358825, 19527078, 257471003, 18252782 |
|  | GO:0050878~regulation of body fluid levels | 4 | 5.9 | 3.0E-02 | 0.453 | 160358825, 19527078, 257471003, 18252782 |
| 1.9 | GO:0030193~regulation of blood coagulation | 3 | 28.0 | 4.9E-03 | 0.184 | 160358825, 257471003, 226958456 |
|  | GO:0032101~regulation of response to external stimulus | 4 | 5.1 | 4.3E-02 | 0.538 | 160358825, 126518317, 257471003, 226958456 |
| 1.8 | GO:0051186~cofactor metabolic process | 6 | 4.3 | 1.2E-02 | 0.296 | 162417975, 33859640, 160358829, 10092608, 29293809, 160298213 |
|  | GO:0006732~coenzyme metabolic process | 5 | 4.6 | 2.3E-02 | 0.387 | 162417975, 33859640, 10092608, 29293809, 160298213 |
|  | GO:0006518~peptide metabolic process | 3 | 10.3 | 3.4E-02 | 0.476 | 162417975, 10092608, 160298213 |
|  | GO:0006790~sulfur metabolic process | 3 | 4.2 | 1.6E-01 | 0.849 | 162417975, 10092608, 160298213 |
| 1.6 | GO:0005976~polysaccharide metabolic process | 5 | 7.1 | 5.2E-03 | 0.180 | 24418919, 226874935, 6755256, 226531047, 31560771 |
|  | GO:0030203~glycosaminoglycan metabolic process | 3 | 9.8 | 3.7E-02 | 0.487 | 226874935, 226531047, 31560771 |
| 1.6 | GO:0034097~response to cytokine stimulus | 4 | 16.3 | 1.8E-03 | 0.079 | 86476056, 130503301, 76881807, 148747546 |
|  | GO:0009725~response to hormone stimulus | 4 | 3.2 | 1.3E-01 | 0.810 | 86476056, 130503301, 76881807, 148747546 |
| 1.5 | GO:0031667~response to nutrient levels | 5 | 5.7 | 1.1E-02 | 0.290 | 15617203, 163310765, 218156289, 6680618, 18252782 |
|  | GO:0009991~response to extracellular stimulus | 5 | 4.9 | 1.8E-02 | 0.347 | 15617203, 163310765, 218156289, 6680618, 18252782 |
| 1.5 | GO:0002526~acute inflammatory response | 7 | 11.3 | 3.3E-05 | 0.003 | 20330802, 130503301, 218156289, 76881807, 110347406, 126518317, 6679182 |
|  | GO:0006952~defense response | 10 | 2.9 | 6.9E-03 | 0.223 | 15617203, 20330802, 130503301, 218156289, 6754976, 76881807, 110347406, 126518317, 19482160, 6679182 |
|  | GO:0006958~complement activation, classical pathway | 3 | 13.1 | 2.2E-02 | 0.378 | 218156289, 110347406, 126518317 |
|  | GO:0048584~positive regulation of response to stimulus | 5 | 3.5 | 5.3E-02 | 0.591 | 163310765, 160358829, 218156289, 110347406, 126518317 |
|  | GO:0050778~positive regulation of immune response | 4 | 3.8 | 8.4E-02 | 0.716 | 160358829, 218156289, 110347406, 126518317 |
|  | GO:0051604~protein maturation | 3 | 4.1 | 1.6E-01 | 0.849 | 218156289, 110347406, 126518317 |
|  | GO:0006955~immune response | 6 | 1.7 | 2.9E-01 | 0.954 | 86476056, 218156289, 6754976, 110347406, 126518317, 6755987 |
| 1.4 | GO:0015669~gas transport | 4 | 40.2 | 1.1E-04 | 0.008 | 157951596, 31982300, 145301578, 21359820, 17647499 |
|  | GO:0015671~oxygen transport | 3 | 35.6 | 3.0E-03 | 0.122 | 31982300, 145301578, 21359820, 17647499 |
|  | GO:0030097~hemopoiesis | 3 | 1.6 | 5.7E-01 | 0.999 | 31982300, 145301578, 21359820, 17647499 |
|  | GO:0002520~immune system development | 3 | 1.3 | 6.6E-01 | 1.000 | 31982300, 145301578, 21359820, 17647499 |
| 1 | GO:0051028~mRNA transport | 3 | 6.3 | 8.0E-02 | 0.709 | 262359885, 163954948, 51712358 |
|  | GO:0006403~RNA localization | 3 | 5.9 | 9.2E-02 | 0.719 | 262359885, 163954948, 51712358 |
|  | GO:0005996~monosaccharide metabolic process | 4 | 2.7 | 1.8E-01 | 0.864 | 33859640, 24418919, 6754450, 6755256 |
| 0.6 | GO:0042592~homeostatic process | 11 | 2.5 | 1.3E-02 | 0.300 | 157951676, 20330802, 6754976, 257471003, 6755256, 6679803, 145301578, 6755114, 77682555, 21359820, 160298213 |
| 0.5 | GO:0042981~regulation of apoptosis | 6 | 1.4 | 4.1E-01 | 0.989 | 113461998, 6679078, 163310765, 6754976, 257471003, 6678101 |
| 0.4 | GO:0032268~regulation of cellular protein metabolic process | 5 | 2.3 | 1.6E-01 | 0.853 | 262359885, 160358829, 31982300, 51712358, 6679803, 17647499 |
| 0.1 | GO:0016265~death | 3 | 0.8 | 9.1E-01 | 1.000 | 86476056, 262359885, 257471003 |

* ES, group enrichment score; ** FE, fold enrichment

**Supplementary Table 2. Clusters of processes from GO FAT database associated with down-regulation by bNOS of protein levels in lymph of challenged mice**

| **ES*** | **Term** | **Count** | **FE**** | **P Value** | **Benjamini** | **Genes** |
| --- | --- | --- | --- | --- | --- | --- |
| 11.71 | GO:0046164~alcohol catabolic process | 15 | 20.8 | 8.2E-15 | 1.1E-11 | 6678674, 124486895, 114326546, 254540027, 6679937, 31982186, 6679261, 70778976, 161484634, 226958349, 13384778, 257743039, 226246531, 6753966, 9256624 |
|  | GO:0044275~cellular carbohydrate catabolic process | 14 | 21.0 | 7.1E-14 | 4.6E-11 | 6678674, 124486895, 114326546, 254540027, 6679937, 31982186, 6679261, 70778976, 226958349, 13384778, 257743039, 226246531, 6753966, 9256624 |
|  | GO:0006007~glucose catabolic process | 13 | 22.5 | 3.0E-13 | 1.3E-10 | 6678674, 124486895, 114326546, 254540027, 6679937, 31982186, 6679261, 70778976, 226958349, 13384778, 257743039, 226246531, 9256624 |
|  | GO:0006091~generation of precursor metabolites and energy | 21 | 7.2 | 8.8E-12 | 1.6E-09 | 227500281, 6678674, 31980762, 6679937, 269973935, 110347487, 268836255, 226958349, 38142460, 257743039, 226246531, 9256624, 18079339, 254540027, 114326546, 160298209, 19705578, 31982186, 6679261, 70778976, 6755911 |
| 4.02 | GO:0030036~actin cytoskeleton organization | 14 | 7.6 | 3.4E-08 | 4.0E-06 | 6755040, 14192922, 10946578, 6753262, 125347376, 6671672, 6680924, 31543113, 114326446, 83921618, 55742711, 6753492, 157951604, 6679601 |
|  | GO:0030029~actin filament-based process | 14 | 7.2 | 7.3E-08 | 7.8E-06 | 6755040, 14192922, 10946578, 6753262, 125347376, 6671672, 6680924, 31543113, 114326446, 83921618, 55742711, 6753492, 157951604, 6679601 |
|  | GO:0007010~cytoskeleton organization | 18 | 5.0 | 1.1E-07 | 1.1E-05 | 6755040, 14192922, 10946578, 6753262, 6755714, 125347376, 6671672, 6754508, 6680924, 31543113, 114326446, 83921618, 55742711, 6753492, 153792001, 45597447, 6679601, 157951604 |
|  | GO:0008064~regulation of actin polymerization or depolymerization | 8 | 14.4 | 1.2E-06 | 1.0E-04 | 6755040, 6753262, 10946578, 6753492, 160837788, 6671672, 6680924, 21312654 |
|  | GO:0030832~regulation of actin filament length | 8 | 14.1 | 1.3E-06 | 1.0E-04 | 6755040, 6753262, 10946578, 6753492, 160837788, 6671672, 6680924, 21312654 |
|  | GO:0032970~regulation of actin filament-based process | 8 | 11.8 | 4.6E-06 | 2.8E-04 | 6755040, 6753262, 10946578, 6753492, 160837788, 6671672, 6680924, 21312654 |
| 3.99 | GO:0006732~coenzyme metabolic process | 11 | 6.9 | 4.1E-06 | 2.8E-04 | 124486895, 31980762, 254540027, 18079339, 269973935, 110347487, 31982186, 149247503, 45597447, 226958349, 13384778 |
|  | GO:0051186~cofactor metabolic process | 12 | 5.9 | 5.5E-06 | 3.1E-04 | 113680352, 124486895, 31980762, 254540027, 18079339, 269973935, 110347487, 31982186, 149247503, 45597447, 226958349, 13384778 |
|  | GO:0006099~tricarboxylic acid cycle | 5 | 19.6 | 1.1E-04 | 4.5E-03 | 254540027, 18079339, 269973935, 110347487, 31982186 |
|  | GO:0009060~aerobic respiration | 5 | 16.7 | 2.1E-04 | 8.0E-03 | 254540027, 18079339, 269973935, 110347487, 31982186 |
|  | GO:0045333~cellular respiration | 6 | 9.2 | 4.8E-04 | 1.5E-02 | 31980762, 254540027, 18079339, 269973935, 110347487, 31982186 |
| 3.35 | GO:0045454~cell redox homeostasis | 8 | 11.6 | 5.1E-06 | 3.0E-04 | 6753086, 13386060, 6671549, 6755911, 31981269, 18017602, 112293264, 110224447 |
|  | GO:0042592~homeostatic process | 17 | 2.6 | 7.3E-04 | 2.2E-02 | 6753136, 31980762, 6753086, 110347487, 84871986, 255760028, 112293264, 14149635, 160298209, 6753492, 6671549, 13386060, 45597447, 6755911, 31981269, 18017602, 110224447 |
|  | GO:0006979~response to oxidative stress | 6 | 6.2 | 2.7E-03 | 6.1E-02 | 31980762, 6753136, 45597447, 6671549, 84871986, 110224447 |
| 2.65 | GO:0006732~coenzyme metabolic process | 11 | 6.9 | 4.1E-06 | 2.8E-04 | 124486895, 31980762, 254540027, 18079339, 269973935, 110347487, 31982186, 149247503, 45597447, 226958349, 13384778 |
|  | GO:0006733~oxidoreduction coenzyme metabolic process | 4 | 9.5 | 8.4E-03 | 1.5E-01 | 124486895, 254540027, 226958349, 13384778 |
| 1.93 | GO:0046907~intracellular transport | 13 | 2.7 | 3.0E-03 | 6.5E-02 | 124487358, 31543974, 125347376, 226874906, 38604071, 6756041, 114326446, 6753364, 153792001, 8567340, 71043944, 148747526, 6678365 |
|  | GO:0008104~protein localization | 17 | 2.0 | 9.0E-03 | 1.6E-01 | 6755040, 124487358, 31543974, 10946578, 29243942, 226874906, 125347376, 38604071, 9507079, 6756041, 114326446, 6753364, 71043944, 8567340, 160333304, 148747526, 6678365 |
|  | GO:0006886~intracellular protein transport | 9 | 2.9 | 1.2E-02 | 1.9E-01 | 124487358, 31543974, 226874906, 71043944, 8567340, 38604071, 6756041, 148747526, 6678365 |
|  | GO:0045184~establishment of protein localization | 13 | 1.8 | 5.9E-02 | 5.4E-01 | 124487358, 31543974, 29243942, 125347376, 226874906, 38604071, 9507079, 6756041, 114326446, 8567340, 71043944, 148747526, 6678365 |
|  | GO:0015031~protein transport | 12 | 1.7 | 1.1E-01 | 7.0E-01 | 124487358, 114326446, 31543974, 29243942, 226874906, 71043944, 8567340, 38604071, 9507079, 6756041, 148747526, 6678365 |
| 1.75 | GO:0043534~blood vessel endothelial cell migration | 3 | 45.0 | 1.8E-03 | 4.7E-02 | 114326446, 84871986, 160333304 |
|  | GO:0016477~cell migration | 8 | 3.0 | 1.7E-02 | 2.5E-01 | 114326446, 6753492, 226874906, 6671672, 6680924, 84871986, 160333304, 157951604 |
| 1.71 | GO:0006575~cellular amino acid derivative metabolic process | 10 | 6.4 | 2.6E-05 | 1.3E-03 | 31980762, 29243942, 62460366, 149247503, 45597447, 262263372, 161484634, 96975138, 6671762, 52353955 |
|  | GO:0006790~sulfur metabolic process | 5 | 4.8 | 2.0E-02 | 2.8E-01 | 31980762, 149247503, 45597447, 262263372, 52353955 |
| 1.48 | GO:0030030~cell projection organization | 10 | 2.8 | 8.9E-03 | 1.6E-01 | 114326446, 6753262, 6753492, 6753364, 45597447, 6671549, 6680924, 96975138, 52353955, 6679601 |
|  | GO:0032989~cellular component morphogenesis | 9 | 2.3 | 4.1E-02 | 4.4E-01 | 114326446, 14192922, 83921618, 6753364, 45597447, 6680924, 96975138, 157951604, 6754222 |
| 1.47 | GO:0006790~sulfur metabolic process | 5 | 4.8 | 2.0E-02 | 2.8E-01 | 31980762, 149247503, 45597447, 262263372, 52353955 |
|  | GO:0006749~glutathione metabolic process | 3 | 11.2 | 2.9E-02 | 3.6E-01 | 31980762, 149247503, 45597447 |
| 1.34 | GO:0050727~regulation of inflammatory response | 4 | 6.3 | 2.5E-02 | 3.3E-01 | 163914390, 7304875, 84871986, 14149635 |
|  | GO:0006800~oxygen and reactive oxygen species metabolic process | 4 | 7.7 | 1.5E-02 | 2.3E-01 | 31980762, 45597447, 84871986, 110224447 |
|  | GO:0003013~circulatory system process | 5 | 4.1 | 3.4E-02 | 4.0E-01 | 31980762, 6671672, 45597447, 12963497, 84871986 |
|  | GO:0042981~regulation of apoptosis | 8 | 1.3 | 4.1E-01 | 9.7E-01 | 7305395, 31980762, 165932331, 45597447, 118130785, 21426847, 84871986, 112293264 |
|  | GO:0006915~apoptosis | 6 | 1.2 | 5.9E-01 | 9.9E-01 | 14192922, 31980762, 46559406, 45597447, 21426847, 84871986 |
|  | GO:0033554~cellular response to stress | 5 | 1.1 | 6.6E-01 | 1.0E+00 | 31980762, 6753086, 45597447, 84871986, 110224447 |
| 1.21 | GO:0050777~negative regulation of immune response | 3 | 10.4 | 3.3E-02 | 3.9E-01 | 163914390, 118130785, 84871986 |
|  | GO:0002683~negative regulation of immune system process | 3 | 3.6 | 2.0E-01 | 8.7E-01 | 163914390, 118130785, 84871986 |
| 1.15 | GO:0009611~response to wounding | 11 | 2.9 | 5.2E-03 | 1.0E-01 | 31980762, 30578393, 163914390, 7304875, 45597447, 118130785, 12963497, 84871986, 6681079, 6753798, 31543113 |
|  | GO:0042060~wound healing | 5 | 4.0 | 3.5E-02 | 4.1E-01 | 30578393, 163914390, 12963497, 84871986, 6753798 |
|  | GO:0007596~blood coagulation | 4 | 5.1 | 4.2E-02 | 4.5E-01 | 30578393, 163914390, 12963497, 6753798 |
|  | GO:0050817~coagulation | 4 | 5.1 | 4.2E-02 | 4.5E-01 | 30578393, 163914390, 12963497, 6753798 |
|  | GO:0007599~hemostasis | 4 | 5.1 | 4.4E-02 | 4.5E-01 | 30578393, 163914390, 12963497, 6753798 |
|  | GO:0050878~regulation of body fluid levels | 4 | 4.0 | 7.5E-02 | 6.1E-01 | 30578393, 163914390, 12963497, 6753798 |
|  | GO:0002526~acute inflammatory response | 3 | 3.3 | 2.3E-01 | 8.8E-01 | 163914390, 7304875, 6753798 |
|  | GO:0006952~defense response | 7 | 1.4 | 3.7E-01 | 9.6E-01 | 213418055, 163914390, 7304875, 118130785, 12963497, 6753798, 110347473 |
|  | GO:0006954~inflammatory response | 4 | 1.6 | 4.5E-01 | 9.8E-01 | 163914390, 7304875, 12963497, 6753798 |
| 0.88 | GO:0008380~RNA splicing | 6 | 2.7 | 7.2E-02 | 6.0E-01 | 116517301, 19527048, 113205059, 83816893, 9790069, 56682933 |
|  | GO:0006397~mRNA processing | 6 | 2.1 | 1.6E-01 | 8.2E-01 | 116517301, 19527048, 113205059, 83816893, 9790069, 56682933 |
| 0.82 | GO:0051248~negative regulation of protein metabolic process | 4 | 4.6 | 5.5E-02 | 5.2E-01 | 226874906, 125347376, 163914390, 118130785 |
|  | GO:0032269~negative regulation of cellular protein metabolic process | 3 | 3.7 | 1.9E-01 | 8.5E-01 | 226874906, 163914390, 118130785 |
|  | GO:0010605~negative regulation of macromolecule metabolic process | 8 | 1.4 | 3.3E-01 | 9.4E-01 | 226874906, 125347376, 163914390, 118130785, 6755911, 14149635, 6755100, 6754222 |
| 0.67 | GO:0006979~response to oxidative stress | 6 | 6.2 | 2.7E-03 | 6.1E-02 | 31980762, 6753136, 45597447, 6671549, 84871986, 110224447 |
|  | GO:0048878~chemical homeostasis | 6 | 1.5 | 3.8E-01 | 9.6E-01 | 31980762, 6753136, 160298209, 110347487, 45597447, 14149635 |
|  | GO:0030003~cellular cation homeostasis | 3 | 1.8 | 4.9E-01 | 9.8E-01 | 6753136, 110347487, 45597447 |
| 0.59 | GO:0045087~innate immune response | 4 | 3.4 | 1.1E-01 | 7.2E-01 | 213418055, 163914390, 118130785, 110347473 |
|  | GO:0006952~defense response | 7 | 1.4 | 3.7E-01 | 9.6E-01 | 213418055, 163914390, 7304875, 118130785, 12963497, 6753798, 110347473 |
|  | GO:0002252~immune effector process | 3 | 2.1 | 4.1E-01 | 9.7E-01 | 213418055, 163914390, 118130785 |
| 0.56 | GO:0043086~negative regulation of catalytic activity | 4 | 3.6 | 1.0E-01 | 6.9E-01 | 125347376, 118130785, 160333304, 14149635 |
|  | GO:0051338~regulation of transferase activity | 5 | 2.3 | 1.8E-01 | 8.4E-01 | 6753364, 45597447, 118130785, 160333304, 14149635 |
|  | GO:0042325~regulation of phosphorylation | 6 | 1.9 | 2.2E-01 | 8.8E-01 | 6753364, 45597447, 118130785, 160333304, 255760028, 14149635 |
|  | GO:0043549~regulation of kinase activity | 4 | 1.9 | 3.6E-01 | 9.5E-01 | 6753364, 45597447, 118130785, 14149635 |
|  | GO:0051347~positive regulation of transferase activity | 3 | 2.0 | 4.4E-01 | 9.8E-01 | 6753364, 45597447, 160333304 |
|  | GO:0045859~regulation of protein kinase activity | 3 | 1.5 | 6.1E-01 | 9.9E-01 | 45597447, 118130785, 14149635 |
|  | GO:0070663~regulation of leukocyte proliferation | 3 | 3.3 | 2.3E-01 | 8.9E-01 | 7305395, 6753492, 118130785 |
| 0.5 | GO:0030334~regulation of cell migration | 3 | 2.9 | 2.7E-01 | 9.1E-01 | 10946578, 6753492, 7304963 |
|  | GO:0048584~positive regulation of response to stimulus | 4 | 1.9 | 3.4E-01 | 9.5E-01 | 7305395, 163914390, 118130785, 14149635 |
|  | GO:0002684~positive regulation of immune system process | 4 | 1.7 | 4.0E-01 | 9.7E-01 | 7305395, 6753492, 163914390, 118130785 |
|  | GO:0050778~positive regulation of immune response | 3 | 2.0 | 4.4E-01 | 9.8E-01 | 7305395, 163914390, 118130785 |
| 0.41 | GO:0010627~regulation of protein kinase cascade | 4 | 2.3 | 2.4E-01 | 9.0E-01 | 125347376, 118130785, 84871986, 255760028 |
|  | GO:0010740~positive regulation of protein kinase cascade | 3 | 3.1 | 2.5E-01 | 9.0E-01 | 125347376, 84871986, 255760028 |
|  | GO:0009967~positive regulation of signal transduction | 3 | 1.6 | 5.7E-01 | 9.9E-01 | 125347376, 84871986, 255760028 |
|  | GO:0010647~positive regulation of cell communication | 3 | 1.4 | 6.2E-01 | 1.0E+00 | 125347376, 84871986, 255760028 |

* ES, group enrichment score; ** FE, fold enrichment
